# Comprehensive Landscape of ARID Family Members and Their Association with Prognosis and Tumor Microenvironment in Hepatocellular Carcinoma

**DOI:** 10.1155/2022/1688460

**Published:** 2022-03-30

**Authors:** Ji Sun, Nan-sheng Cheng

**Affiliations:** Department of Biliary Surgery, West China Hospital of Sichuan University, Chengdu 614100, Sichuan Province, China

## Abstract

As one of the most lethal forms of cancers, hepatocellular carcinoma (HCC) claims many lives around the world, and it is especially common in China. The ARID family plays key roles in the pathogenesis and development of human cancers. The potential of several functional genes used as novel biomarkers has attracted more and more attention. However, the prognostic values of the ARID family in HCC patients are rarely known by people. In this study, we performed comprehensive analysis using TCGA datasets, finding that the expressions of ARID4B, ARID2, ARID3B, JARID2, ARID1A, ARID1B, and ARID3A were increased in HCC specimens compared to nontumor specimens, while the expressions of ARID4A and ARID3C were decreased in HCC specimens. According to the Pearson correlation data, the methylation levels of the majority of ARID members were negatively correlated. Upregulation of ARID3A, ARID5B, and ARID1A was related to a poor HCC outcome according to the data of multivariate assays. Then, we built a LASSO Cox regression model based on ARID3A, ARID5B, and ARID1A in HCC. Overall survival rates were considerably lower for those with high risk scores compared to those with low risk scores. Finally, we studied the associations between risk scores and several types of infiltrating immune cells. The data revealed that the risk score was positively related to the infiltration of CD8+ T cells, B cell, T cell CD8+, neutrophil, macrophage, and myeloid dendritic cell. This study conducted a thorough analysis of the ARID members, resulting in new insights for further examination of the ARID family members as prospective targets in the treatment of HCC.

## 1. Introduction

Especially in Asian and African populations, hepatocellular carcinoma (HCC) is the third greatest cause of cancer death worldwide, coming in at number five behind lung and breast cancer [1, 2]. Approximately 1 million individuals are predicted to die each year from HCC since this disease is so easily confused with cirrhosis, making early detection difficult and leading to a dramatic rise in HCC patients [3]. Despite endeavors in the field of science and tremendous advances in understanding the fundamental molecular event in HCC, 5-year survival rates have not altered significantly in the last few years because of the lack of information on the causes of liver cancer development [4, 5]. In order to correctly forecast the progression and prognosis of HCC, useful biomarkers must be developed as soon as possible.

The human AT-rich interaction domain (ARID) family is a superfamily of fifteen members, containing JARID2, JARID1D, JARID1C, JARID1B, JARID1A, ARID5B, ARID5A, ARID4B, ARID4A, ARID3C, ARID3B, ARID3A, ARID2, ARID1B, and ARID1A [6, 7]. Subfamilies are formed depending on the degree of sequence identified between each member of the families [8, 9]. While interacting with AT-rich DNA elements, members of the ARID family were identified to show a DNA-binding domain, which was the first time it had been observed [10]. Researchers have discovered that ARID genes act as transcription regulators and can influence cell growth and differentiation [11, 12]. It has recently been discovered through a rising number of research investigations that the ARID family of proteins might be implicated as a factor in the development of human cancer [13, 14]. For instance, through its impact on promyelocytic leukemia's stability, ARID3A acted as a significant antagonist to the p16 retinoblastoma protein inhibitor mechanism [15]. Silence of ARID1A had been linked to an epithelial-mesenchymal transition progress and increased sensitivity of pancreatic tumor cells to NVP-AUY922, a therapeutic inhibitor of HSP90 according to a prior study [16]. Although ARID family members have been implicated in several types of neoplasms, according to a number of studies, the expressions and prognostic significances of each ARID, particularly at the mRNA levels in HCC, remained unclear and complex.

By analyzing TCGA datasets, we were able to determine ARID mRNA expressions in HCC specimens and normal specimens. Then, we aimed to determine the predictive significance of each member of ARID expressions in HCC cases using Kaplan-Meier assays. We discovered a number of indicators that were previously unreported in patients with HCC. The investigations served as an excellent starting point for the subsequent study.

## 2. Materials and Methods

### 2.1. Data Collection

Three hundred seventy-one HCC patients had their level three RNA sequencing (RNA-seq) data and accompanying clinical information obtained from TCGA website up until July 10, 2020. The “limma” R package's scale methods were used to normalize the gene expression patterns [17]. TCGA's data is available to the general public. As a result, local ethics committees were not required to approve this study. The current study adhered to TCGA's data-sharing and publication policies and procedures.

### 2.2. Analysis of ARID Family Members' Expression Levels in HCC

We first analyzed the expression levels of 15 members containing JARID2, JARID1D, JARID1C, JARID1B, JARID1A, ARID5B, ARID5A, ARID4B, ARID4A, ARID3C, ARID3B, ARID3A, ARID2, ARID1B, and ARID1A, in HCC specimens and nontumor specimens. TPM-normalized expression levels were used for RNA-seq analysis. The Wilcoxon test proved the significance of the two sets of samples. In order to be declared statistically significant, *p* values of less than 0.05 were required.

### 2.3. ARID Family Members' mRNA Expression and Methylation in HCC Have a Corresponding Relationship

Gene promoter areas of ARID family members that were differentially expressed in HCC tissues were downloaded from the Illumina HumanMethylation 450K using GDC Data Transfer Tool, which was recommended by TCGA for this purpose. After that, we used the corrplot tool to investigate the association between ARID expressions and methylation in HCC further.

### 2.4. Research into the Predictive Gene Signature and Its Reliability

Using the “glmnet” package for R, the LASSO assays were applied to construct multivariable models containing genes from the ARID gene family [18]. In order to achieve dimension reduction of high-dimensional data, the LASSO regression technique restricts the sum of the absolute values of the coefficients to be smaller than a predefined value. Consequently, a coefficient of zero would be assigned to variables with a small contribution. Maximizing model performance while limiting the amount of features led to the best model. For the purpose of calculating the risk score, only genes with nonzero coefficients in the LASSO regression model were selected for further consideration. A threshold value of the median risk score was used to categorize all of the patients in the study.

### 2.5. Immune Cell Infiltration Was Analyzed Using Association Analysis

Immune infiltration data of neutrophils, macrophages, dendritic cells, CD8+ T cells, CD4+ T cells, and B cells were provided by the tumor immune estimation resource (TIMER) database [19]. Pearson's correlation study examined the link between immune infiltration risk ratings.

### 2.6. Statistical Analysis

HTSeq FPKM and methylation data were extracted using Perl 5.32 software. R x64 4.0.5 software and open-source websites were used for bioinformatics statistical analysis. A *t*-test was used to compare the findings of two separate groups. For the Kaplan-Meier, time-dependent ROC curve, univariate, and multivariate assays, we used R packages “survival” and “survivalROC” to compare the survival rates of the cohort's high- and low-risk groups. All of the *p* values in this study were below the threshold for statistical significance of 0.05.

## 3. Results

### 3.1. Differential Expression of FXYD Family Member Genes in HCC

**Figure 1:**
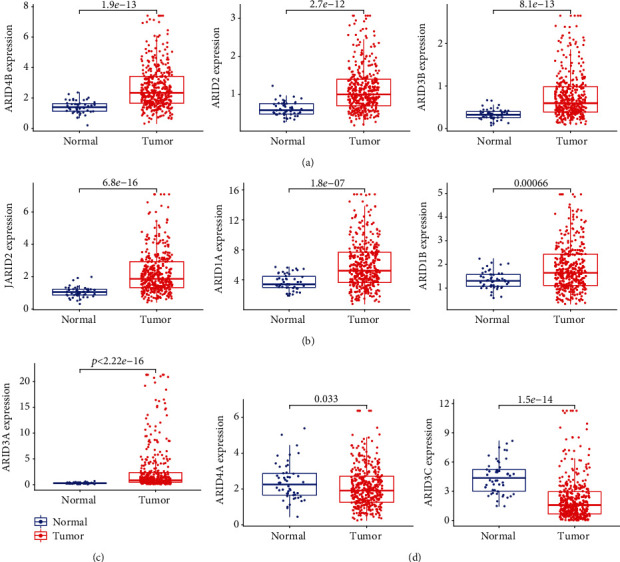
Differential expression analysis of ARID family members in HCC. (a) ARID4B, ARID2, and ARID3B; (b) JARID2, ARID1A, and ARID1B; and (c) ARID3A were significantly overexpressed in HCC. (d) ARID4A and ARID3C were significantly downregulated in HCC.

To identify the abnormal expression of FXYD family member genes in HCC, we analyzed TCGA datasets, finding that ARID4B, ARID2, ARID3B, JARID2, ARID1A, ARID1B, and ARID3A were increased in HCC tissues compared to nontumor specimens (Figures 1(a)–1(c)), while ARID4A and ARID3C were decreased in HCC tissues compared to nontumor specimens (Figure 1(d)).

### 3.2. Correlation of HOXA Expression and Methylation in HCC

**Figure 2:**
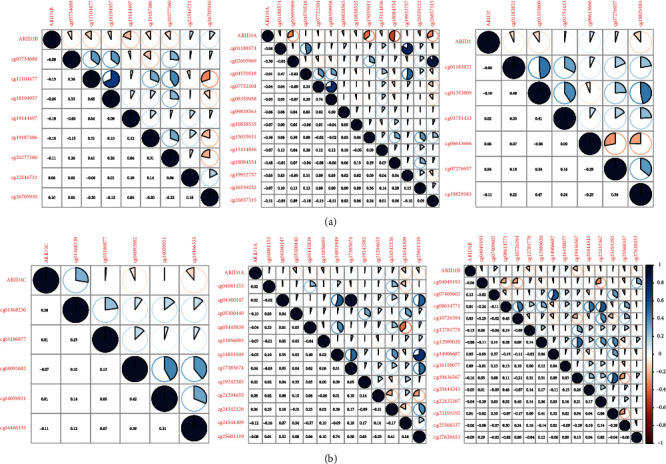
Pearson's correlation between methylation levels and expression of (a) ARID3B, ARID3A and ARID2 and (b) ARID3C, ARID1A, and ARID1B.

It is one of the most common processes by which gene expression is influenced during the course of human tumors that methylation of gene promoter regions occurred. We identified seven differentially expressed FXYD family member in HCC (downregulated ARID4A and ARID3C and upregulated ARID4B, ARID2, ARID3B, JARID2, ARID1A, ARID1B, and ARID3A). Many members of the FXYD family had a negative connection with methylation level, according to Pearson's correlation data. (Figures 2(a) and 2(b) and Figure S1).

### 3.3. The Prognostic Values of Members of the FXYD Family in Individuals with HCC

**Figure 3:**
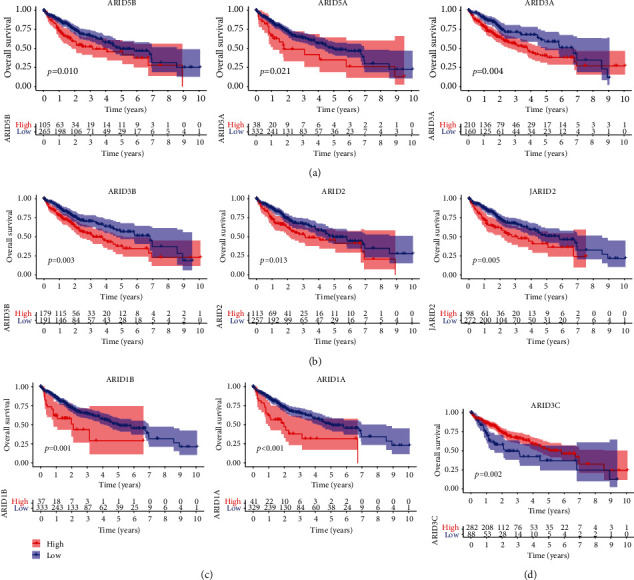
Prognostic values of ARID members in overall survival of all HCC patients. Survival assays related that (a) ARID5B, ARID5A, and ARID3A; (b) ARID3B, ARID2, and JARID2; and (c) ARID1B and ARID1A predicted a shorter overall survival in HCC patients. (d) Patients with high ARID3C expression exhibited a longer overall survival.

**Figure 4:**
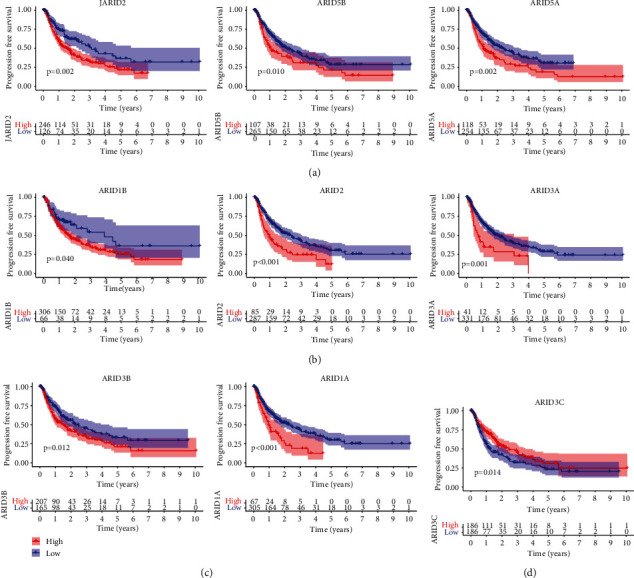
Prognostic values of ARID members in progression-free survival of all HCC patients. Survival assays related that (a) JARID2, ARID5B, and ARID5A; (b) ARID1B, ARID2, and JARID3A; and (c) JARID3B and ARID1A predicted a shorter overall survival in HCC patients. (d) Patients with high ARID3C expression exhibited a longer overall survival.

**Figure 5:**
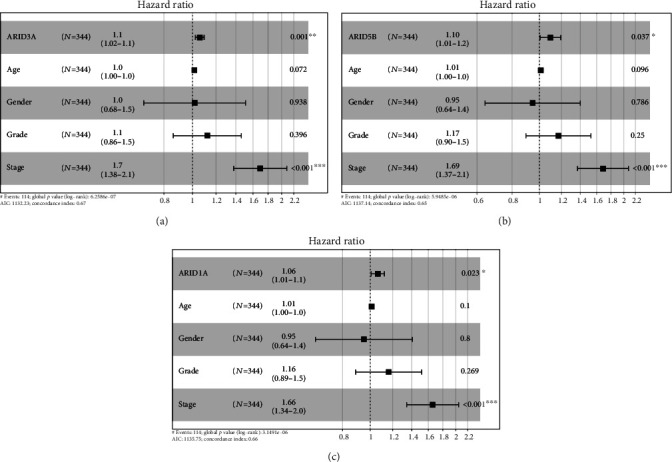
The results of multivariate assays of important prognostic factors are shown in forest plots: ARID3A (a), ARID5B (b), and ARID1A (c).

After that, we ran Kaplan-Meier assays to determine the clinical importance of FXYD family members in HCC patients. We found that high expressions of ARID1A, ARID1B, ARID3A, ARID2, ARID3B, ARID5A, ARID5B, and JARID2 predicted a shorter overall survival in HCC patients (Figures 3(a)–3(c)), while overall survival was reduced in patients with high ARID3C expression compared to low ARID3C expression. (Figure 3(d)). Moreover, the similar findings were observed using the data of progression free survival (Figures 4(a)–4(d)). The multivariate Cox proportional hazards regression analysis was used to evaluate the independent prognostic values of members of the FXYD family. The findings revealed that the expressions of ARID3A (Figure 5(a)), ARID5B (Figure 5(b)), and ARID1A (Figure 5(c)) were all independent predictive indicators of HCC outcome in the study participants.

### 3.4. Construction of Prognostic Model Based on ARID3A, ARID5B, and ARID1A for HCC

**Figure 6:**
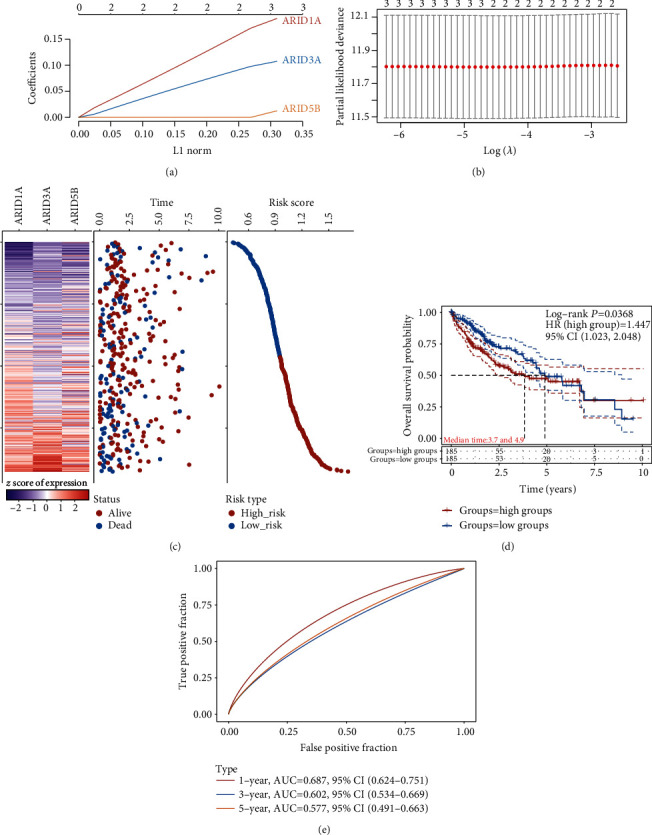
TCGA datasets showed a link between the three-gene signature and patient survival. (a) Threefold cross-validation is used in the LASSO model to identify tuning parameters for tuning. (b) LASSO coefficient profiles of ARID3A, ARID5B, and ARID1A. (c) Distributions of risk scores, survival statuses of patients in low-risk and high-risk groups (middle), and ARID3A, ARID5B, and ARID1A expression profiles of each patient (bottom). (d) Overall survival Kaplan-Meier curves for high- and low-risk groups. (e) ROC curve for the 1, 3, and 5-year survival prediction by the three-gene signature.

The prognostic model consisting of 3 FXYD genes (ARID3A, ARID5B, and ARID1A) was constructed using LASSO regression (Figures 6(a) and 6(b)). The risk score model was created based on the findings of the LASSO and multivariate assays. The prognostic risk score for each patient was computed utilizing the 3-gene signature prognostic model. The median scores were applied to split HCC patients into low-risk and high-risk categories. As demonstrated in Figure 6(c), there was a significant difference in risk score, survival status, and expression pattern between groups at high and low risk. Prognosis for the two groups differed significantly according to Kaplan-Meier curve data (*p* < 0.001). The high-risk group's prognosis was much worse than the low-risk group's (Figure 6(d)). In patients with HCC, the 3-gene signature prognostic model performed well in predicting prognosis, as evidenced by the model's AUC values of 0.687, 0.602, and 0.577 for 1-, 3-, and 5-year OS (Figure 6(e)).

### 3.5. Assessment of the Relationship between the Tumor-Infiltrating Immune Cells and the Risk Score

**Figure 7:**
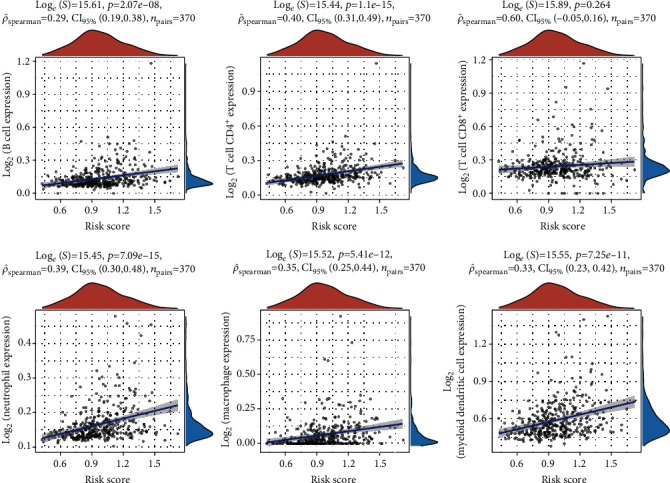
Correlation of infiltrating immune cells with risk score. Scatter plot indicated the correlations of six kinds of infiltrating immune cells with the risk score (*p* < 0.05). Correlation tests were carried out using Pearson coefficients, and the blue line in each plot was fitted linear model demonstrating immune cell tropism together with risk score.

We analyzed the correlation between risk score and several types of infiltrating immune cells. The results showed that the risk score was positively related to the infiltration of CD8+ T cells, B cell, T cell CD8+, neutrophil, macrophage, and myeloid dendritic cell (Figure 7). These findings may potentially provide more evidence that our model was accurate in predicting the outcome of HCC. Further investigation into the relationships between risk score and the six types of invading immune cells is required to corroborate these findings.

## 4. Discussion

A growing variety of innovative treatment methods for HCC are being developed, including immune therapy, gene therapy, and molecular targeted therapy [20, 21]. However, HCC still has a poor prognosis due to the lack of successful therapeutic interventions. Novel prognostic and therapeutic methods aiming at improving the prognosis of patients with HCC require a thorough understanding of the molecular pathways underlying tumor genesis and progression [22, 23]. Multiple cancers have been linked to members of the ARID family, which may be involved in carcinogenesis and prognosis; however, they need to be studied in future HCC studies for their oncological and prognostic significance.

Previous studies have demonstrated that the ARID family of genes contributed to the development of human malignancies according to expression data. For instance, tumor suppressor activity is correlated with reduced ARID1A expression in CRC tissues [24]. It was discovered in 91 cases that ARID3A positive was associated with absence of perineural invasion, longer disease-free life, and longer cancer-specific survival [25]. Huang and his group reported that comparing CRC tissue to nearby normal tissues, JARID1B was highly elevated. A high level of JARID1B expression was linked to a poor prognosis in CRC patients [26]. However, the expression of the entire ARID family in HCC was not previously comprehensively investigated. ARID4A and ARID3C were downregulated in HCC samples compared with normal samples in this in silico analysis, which established the expression profile of ARID members in HCC. In contrast, ARID4B, ARID2, ARID3B, JARID2, ARID1A, ARID1B, and ARID3A were increased in HCC specimens compared to nontumor specimens.

Up to this point, DNA methylation events have been found to be the most significant and frequent epigenetic modifications in mammalian genomes [27, 28]. The downregulation of LINC00261 by DNA hypermethylation is critical for the development of LADCs because it stimulates their proliferation and survival [29]. To explore the possible mechanisms involved in the abnormal expression of ARID members in HCC, we downloaded methylation datasets from TCGA datasets. According to the results of the Pearson correlation, among the nine differentially expressed ARID members (ARID4B, ARID2, ARID3B, JARID2, ARID1A, ARID1B, ARID3A, ARID4A, and ARID3C), most expression levels are affected by the methylation level.

Previously, the function and prognostic value of ARID members have been reported in several tumors, including HCC. For instance, Cheng et al. reported that ARID1A inhibits HCC cell proliferation and migration by upregulating its downstream target [30]. Compared to nearby normal liver tissues, ARID4B was shown to be strongly expressed in HCC tissues. A poor prognosis for HCC patients was indicated by high ARID4B expression, which was found to be associated with tumor-node-metastasis stages, Edmondson-Steiner grades, vascular invasion, and tumor size [31]. According to these findings, ARID members have the potential to be exploited as novel biomarkers for HCC diagnosis. In this study, we firstly explored the prognostic value of the whole ARID members and found that the expressions of JARID2, ARID5B, ARID5A, ARID3C, ARID3B, ARID3A, ARID2, ARID1B, and ARID1A were related to overall survival and progression-free survival of HCC patients. More importantly, based on the results of COX assays, we confirmed that ARID3A, ARID5B, and ARID1A were independent poor prognostic factors for 5-year overall survival of HCC patients.

In recent years, more and more prognostic model based on multiple genes was developed [32, 33]. For instance, Cao et al. developed the discovery of a unique EMT-related gene signature in bladder cancer that exhibited tumor-promoting effects, operated as a negative independent prognostic factor, and may facilitate individualized counseling and therapy in this disease [34]. Zhou and his group identified a set of six lncRNAs (XLOC_004803, AC073115.6, RP11-89C21.2, ENTPD1-AS1, UBE2R2-AS1, and AC005013.5) which can be used as a novel marker for glioma patients. The six-lncRNA signature may be engaged in immune-related biological processes and pathways that are well-known in the setting of glioblastoma carcinogenesis [35]. In this study, we developed a three-gene signature using ARID3A, ARID5B, and ARID1A. Patients were separated into two groups based on the new signature: those at high risk and those at low risk. The prognosis was worse for those patients who were classified as high risk. Analysis of the ROC curve indicated that the metabolic signature had a good predictive accuracy.

Previous researches have shown that HCC patients' immune systems play a critical role in their responsiveness to treatment and prognosis [36, 37]. Individual immune cell indicators have been found to affect the prognosis of HCC patients [38, 39]. PD-L1 on mesenchymal stromal cells may promote HCC by suppressing CD8+ T cell antitumor immune responses, according to previous studies that found that inhibiting CD8+ T cells accelerated tumor progression [40, 41]. In the present study, the risk score was found to be associated with the infiltration of myeloid dendritic cells, macrophages, neutrophils, CD8+ T cells, B cells, and CD8+ T cells. These findings may also support our model's ability to accurately forecast the prognosis of HCC patients.

There are some limitations to this study that warrant consideration. Firstly, our data were downloaded from solely TCGA datasets; the sample size is relatively small. Secondly, using RNA-seq data, bioinformatics tools were used to map the immune system's landscape. The results of this study could have been skewed by random noise. In addition, all of the above results need to be verified in basic experiments and clinical trials.

## 5. Conclusion

Our bioinformatics findings show that multiple members of the ARID family exhibited a dysregulated level in HCC. ARID3A, ARID5B, and ARID1A were revealed as independent prognostic factors for HCC patients. For patients with HCC, our work developed a robust predictive signature that might be used to better stratify their risk of death and tailor their treatment to their specific needs. HCC patients' ARID family members are deserving of further study as potential clinical biomarkers.

## Data Availability

The analyzed datasets generated during the study are available from the corresponding author on reasonable request.

## Supplementary Materials

Supplementary Materials Figure S1: Pearson's correlation between methylation levels and expressions of ARID5B, ARID5A, ARID4B, AJRID2, and ARID4A.
