# Supplementary figures and images for: Comprehensive Landscape of ARID Family Members and Their Association with Prognosis and Tumor Microenvironment in Hepatocellular Carcinoma
Source: J Immunol Res. 2022 Mar 30;2022:1688460. doi: 10.1155/2022/1688460 (PMC8986425; doi:10.1155/2022/1688460)

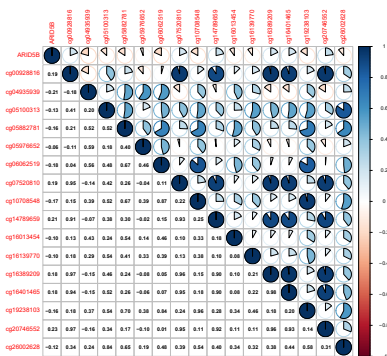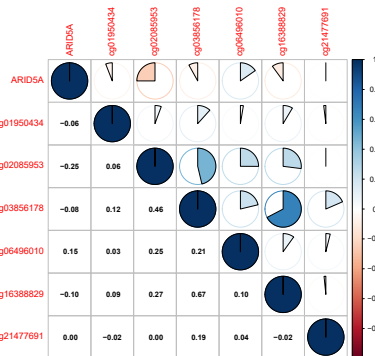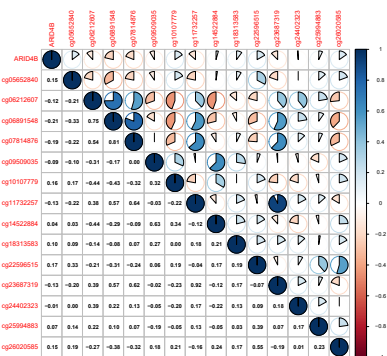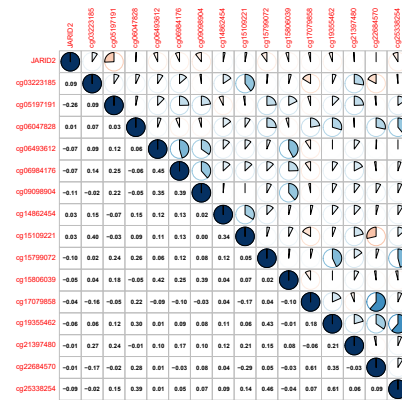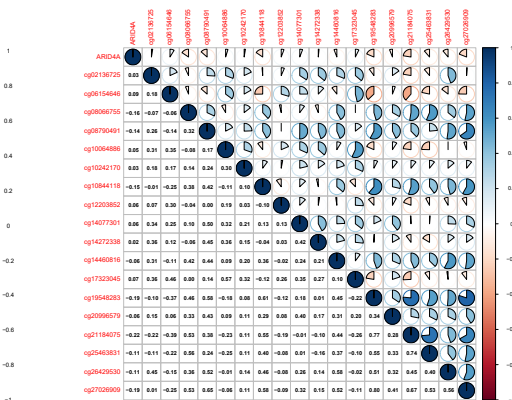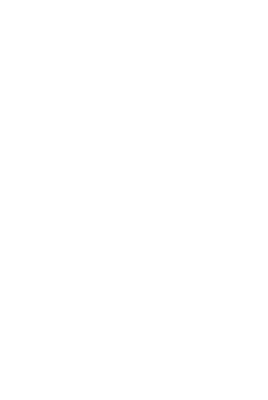

Supplement: Supplementary Materials — Figure S1: Pearson's correlation between methylation levels and expressions of ARID5B, ARID5A, ARID4B, AJRID2, and ARID4A. [file 1688460.f1.pdf]
